# Isolation and Evaluation of Oil-Producing Microalgae from Subtropical Coastal and Brackish Waters

**DOI:** 10.1371/journal.pone.0040751

**Published:** 2012-07-11

**Authors:** David K. Y. Lim, Sourabh Garg, Matthew Timmins, Eugene S. B. Zhang, Skye R. Thomas-Hall, Holger Schuhmann, Yan Li, Peer M. Schenk

**Affiliations:** 1 School of Agriculture and Food Sciences, The University of Queensland, Brisbane, Queensland, Australia; 2 ARC Centre of Excellence in Plant Energy Biology, Centre for Metabolomics, School of Chemistry and Biochemistry, The University of Western Australia, Crawley, Western Australia, Australia; J. Craig Venter Institute, United States of America

## Abstract

Microalgae have been widely reported as a promising source of biofuels, mainly based on their high areal productivity of biomass and lipids as triacylglycerides and the possibility for cultivation on non-arable land. The isolation and selection of suitable strains that are robust and display high growth and lipid accumulation rates is an important prerequisite for their successful cultivation as a bioenergy source, a process that can be compared to the initial selection and domestication of agricultural crops. We developed standard protocols for the isolation and cultivation for a range of marine and brackish microalgae. By comparing growth rates and lipid productivity, we assessed the potential of subtropical coastal and brackish microalgae for the production of biodiesel and other oil-based bioproducts. This study identified *Nannochloropsis* sp., *Dunaniella salina* and new isolates of *Chlorella* sp. and *Tetraselmis* sp. as suitable candidates for a multiple-product algae crop. We conclude that subtropical coastal microalgae display a variety of fatty acid profiles that offer a wide scope for several oil-based bioproducts, including biodiesel and omega-3 fatty acids. A biorefinery approach for microalgae would make economical production more feasible but challenges remain for efficient harvesting and extraction processes for some species.

## Introduction

Interest in a renewable source of biofuels has recently intensified due to the increasing cost of petroleum-based fuel and the dangers of rising atmospheric CO_2_ levels. Among the various candidates for biofuel crops, photosynthetic microalgae have the advantage that they have high growth rates and can be cultured on non-arable land [1], [2], [3].

At present, microalgae are commercially grown at scale for fatty acid-derived nutraceuticals and as feed and food supply. Significant interest in microalgae for oil production is based on their ability to efficiently convert solar energy into triacylglycerides (TAGs), which can be converted to biodiesel via transesterification reactions [1], [4], [5]. Oleaginous microalgae are capable of accumulating 20–50% of their dry cell weight as TAGs and potentially have a productivity superior to terrestrial crops used as first generation biofuel feedstock [6]. Theoretical calculations of microalgal oil production (liter/ha) are 10 to 100-fold greater than traditional biodiesel crops such as palm oil [7], corn and soybeans [6], [8], [9], although large-scale commercial algal oil production has yet to be established. Another major advantage of microalgae over higher plants as a fuel source is their environmental benefits. Despite having to grow in an aquatic medium, microalgae production may require less water than terrestrial oleaginous crops and can make use of saline, brackish, and/or coastal seawater [10], [11]. This allows the production of microalgae without competing for valuable natural resources such as arable land, biodiverse landscapes and freshwater. Furthermore, a microalgae-based biofuel industry has tremendous potential to capture CO_2_. In high efficiency, large microalgae cultivation systems, the potential capture efficiency of CO_2_ can be as high as 99% [12], effectively capturing 1.8 kg of CO_2_ per kg of dry biomass [13]. Although CO_2_ captured this way into biodiesel will eventually be released upon combustion, this would displace the emission of fossil CO_2_ and the remaining biomass (e.g. ∼70% of dry weight) can be fed into downstream carbon sequestration processes. For example, sequestering carbon into hard C-chips (Agri-char) via pyrolysis can be used to improve soil fertility, mitigating climate change by reintroducing durable carbon back into the soil [14], although it is debatable how long this carbon will actually stay in the soil.

Aside from biodiesel production, microalgae are gaining a reputation as “biofactories” due to the varied composition of their biomass. Akin to today's petroleum refinery, which produces a range of fuels and derivative products, a well-managed and equipped microalgal biorefinery can produce biodiesel and other value-add products such as protein, carbohydrates and a range of fatty acids (FAs). High value omega-3 fatty acids (ω-3) such as eicosapentaenoic (EPA), docosahexanoic (DHA), alpha-linolenic acid (ALA) and arachidonic (AA) are not desirable FAs for biodiesel production. Nevertheless, these ω-3 polyunsaturated fatty acids (PUFAs) are highly valued in human nutrition and therapeutics [15] and are linked to a wide range of cardio and circulatory benefits [16]. Ω-3 fatty acids also play an important role in aquaculture, increasing growth performance and reducing mortality in the shellfish industry [17], [18], [19]. This ability to produce value-adding products in addition to biodiesel is important to reduce production cost and make large-scale production viable.

The inherent advantages of a microalgal fuel source are unfortunately offset by current limitations to economically produce it on a large-scale. For example, the cost for obtaining dry biomass, large hexane requirements and limited hexane recycling capacity are currently hindering economic viability. It was estimated that the current cost of producing 1 tonne of microalgal biomass with an average 55% (w/w_DryWeight_) oil content needs to be reduced by 10-fold in order to be competitive with petroleum diesel [8]. Furthermore, despite estimates that suggest microalgal oil production (US$9–$25/gallon in ponds, $15–$40 in photobioreactors) could be cheaper than the current price of oil [20], companies commercially producing microalgae have not been able to achieve the predicted yields and production costs. Typical lipid yields of 10 g m^−2^d^−1^ (Skye Thomas-Hall, personal communication) are still short of achieving the current best case scenarios of 103 to 134 g m^−2^d^−1^ [21]. The industry is still in its infancy, although recent research and development efforts by large oil companies (e.g. Exxon, BP, Chevron and Shell) would certainly increase production capacity and decrease production costs.

As large variations (10–50%) in lipid content exist between different species of microalgae [22], [23], it is necessary to identify strains with high lipid content and suitable lipid composition. The need for high-yielding microalgae is straightforward, as this directly translates to an overall increase in production, although lipid production during normal growth needs to be distinguished from lipid accumulation in response to adverse conditions (e.g. nutrient starvation). Lipid composition is equally important, as quantitative and qualitative differences in the TAG content of a given species will affect the quality of biodiesel and its ability to meet fuel standards. Fuels with high cetane number fatty acids (e.g. myristic acid, palmitic acid, stearic acid) are desirable [24], as higher cetane fuels have better combustion quality and the right cetane number of biodiesel is required to meet an engine's cetane rating [25]. Microalgal lipids are mostly polyunsaturated, which have a low cetane number and are more prone to oxidation. This can create storage problems and are thus preferred to be at a minimum level for biodiesel production. Nevertheless, polyunsaturated fatty acids lower the cold filter plugging point (CFPP) of fuel and are crucial in colder climates to enable the biodiesel to perform at lower temperatures [3]. With these factors in mind, an “ideal composition” of fatty acids would consist of a mix of saturated and monounsaturated short chain fatty acids in order to have a very low oxidative potential whilst retaining a good CFPP rating and cetane number.

To date, research efforts have focused on lipid production of individual species, usually investigating the effects different growth conditions have on lipid production and content [26], [27], [28], [29], [30]. Unfortunately, direct comparisons of results between studies are unreliable, given the different growth conditions and experimental parameters of each species and also the different methods used for lipid extraction. There is growing interest to compare lipid content and FA composition of multiple microalgae species [11], [31], [32], [33], [34], [35]. Several studies have revealed algae genera such as *Tetraselmis, Nannochloropsis* and *Isochrysis* to have highest high lipid content, particularly under nutrient-deprived conditions [11], [31].

Nutrient deprivation is regarded as an efficient way to stimulate lipid production in microalgae in several microalgae species [11], [29], [36], [37], especially saturated and monosaturated FAs [6], [38], [39]. Unfortunately, lipid accumulation is often associated with a reduction in biomass, which reduces overall lipid accumulation. A batch culture strategy can be adopted to obtain maximal biomass productivity as well as induction of lipid accumulation through nutrient deprivation. Although a common research practice, only Rodolfi et al. [11] have published lipid profiles of multiple microalgae species in a batch culture setting.

The target of our work was to identify the most effective microalgal TAG producers for biodiesel production using a basic batch culture strategy. Most studies utilize experimental designs that include aeration of media volumes of 1 L to 10 L in order identify microalgae strains with high lipid content [31], [32], [33], [36], [40]. To provide a direct comparison between different species, this study evaluated eleven microalgae strains collected from local Australian coastal waterways and other collections that originate in various places in the world. Strains were first characterized by microscopy and partial 18S ribosomal RNA sequencing and total fatty acid methyl ester (FAME) contents were then analyzed via GC/MS, which quantifies the fatty acids in triacylglycerides in each strain, thus providing the most accurate representation of the substrate available for biodiesel production. Using growth rate, FAME productivity and FA composition as criteria, this study identified several algae strains to be suitable for biodiesel, including *Tetraselmis* sp. and *Nannochloropsis* sp. as highly versatile candidate strains for a multiple-product algal biorefinery.

## Materials and Methods

### Microalgae strain collection and isolation

Microalgae were collected as 10 mL water samples from coastal rock pools, freshwater lakes and brackish (tidal) riverways. After initial cultivation of the mixed cultures with F medium [41] pure cultures were isolated by performing serial dilutions and the use of a micromanipulator (Leica DMIL with Micromanipulator). Strains *Chlorella* sp. BR2 and *Nannochloropsis* sp. BR2 originated from the same water sample and were collected from the Brisbane river (27°31′21″S 153°0′32″E; high tide at 10 am in August 2007 on a sunny day). Strain *Tetraselmis* sp. M8 was collected in an intertidal rock pool at Maroochydore (26°39′39″S 153°6′18″E; 12 pm on 6 August 2009). Additional, microalgae strains used in this study were obtained from the Australian National Algae Culture Collection (ANACC, CSIRO) and Queensland Sea Scallops Trading Pty Ltd (Bundaberg, Australia) (Table 1). All primary stock cultures were maintained aerobically in 100 mL Erlenmeyer flasks with constant orbital shaking (100 rpm) at 25°C, under a 12∶12 h light/dark photoperiod of fluorescent white light (120 μmol photons m^−2^s^−1^). All cultures except *Chlorella* sp. were grown in seawater complemented with F medium [41]. *Chlorella* sp. was cultured in freshwater complemented with F medium. Primary stock cultures were sub-cultured every 3 weeks to minimize bacterial growth. Non-sterile cultures were used and maintained, as difficulties in maintaining axenic cultures in real production would arise and axenic cultures had been reported to have low biomass productivity, most likely because algae-associated bacteria may assist in nutrient recycling [42]. However, all microalgae cultures were checked during cell counting to ensure that no contamination with other microalgae occurred.

**Table 1:** Sources and 18S rRNA sequence accessions of microalgae strains used in this study.

Species	Genbank Accession	Location of Origin
*Tetraselmis sp.* M8	JQ423158	Maroochydore, Qld, Australia
*Tetraselmis chui*	JQ423150	East Lagoon, Galveston, TX, USA
*Tetraselmis suecica*	JQ423151	Brest, France
*Nannochloropsis* sp. BR2	JQ423160	Brisbane River, Brisbane, Australia
*Dunaliella salina*	JQ423154	Alice Springs, NT, Australia
*Chaetoceros calcitrans*	JQ423152	Unknown
*Chaetoceros. muelleri*	JQ423153	Oceanic Institute, Hawaii, USA
*Pavlova salina*	JQ423155	Sargasso Sea
*Pavlova lutheri*	JQ423159	Unknown location, UK
*Isochrysis galbana*	JQ423157	Unknown location, UK
*Chlorella sp.* BR2	JQ423156	Brisbane River, Brisbane, Australia

### Standard protocol for batch culture growth analysis, lipid induction phase and sampling for lipid analysis

A standard protocol was designed to allow direct comparisons of growth rates and lipid productivity between cultures. To standardize inoculum cell densities, cultures were first grown to late logarithmic phase in F medium. Late-log phase of each culture was determined when daily cell count of the pre-culture revealed a less than 20% increase in cell density. A total of 1 mL of pre-culture in late-log phase was used as inoculum (7 to 9 hours after start of light cycle) for 20 mL seawater (SW) complemented with F medium in 100 mL Erlenmeyer flasks. A minimum of three parallel cultures were grown in conditions as described above. Cell counts were performed on days 0, 2, 4, 6 and 7 post inoculation using a haemocytometer. After day 7, nutrient deprivation to stimulate lipid production was achieved by removal of previous medium by centrifugation (1,200×g, 5 min) and replacement with only SW (without F medium). Cultures were then grown for another 48 h before 4 mL of wet biomass from each replicate was harvested for lipid analyses.

### Fatty Acid Methyl Ester (FAME) analyses

Algae cultures (4 mL each) were centrifuged at 16,000× g for 3 min. The supernatant was discarded and lipids present in the algal pellet were hydrolyzed and methyl-esterified by shaking (1,200 rpm) with 300 µL of a 2% H_2_SO_4_/methanol solution for 2 h at 80°C; 50 µg of heneicosanoic acid (Sigma, USA) was added as internal standard to the pellet prior to the reaction. A total of 300 µL of 0.9% (w/v) NaCl and 300 µL of hexane was then added and the mixture was vortexed for 20 s. Phase separation was performed by centrifugation at 16,000× g for 3 min. A total of 1 µL of the hexane layer was injected splitless into an Agilent 6890 gas chromatograph coupled to a 5975 MSD mass spectrometer. A DB-Wax column (Agilent, 122–7032) was used with running conditions as described for Agilent's RTL DBWax method (Application note: 5988–5871EN). FAMEs were quantified by taking the ratio of the integral of each FAME's total ion current peak to that of the internal standard (50 µg). The molecular mass of each FAME was also factored into the equation. Identification of FAME was based on mass spectral profiles, comparison to standards, and expected retention time from Agilent's RTL DBWax method (Application note: 5988–5871EN).

### DNA isolation and sequencing

Genomic DNA was isolated from all algal species via a phenol-chloroform method [43] on a pellet obtained by centrifugation of 10 mL of algal culture at the late-log phase. DNA amplification from genomic DNA containing a partial 18S ribosomal RNA region was performed by PCR using the following primers: Forward: 5′-GCGGTAATTCCAGCTCCAATAGC–3′ and Reverse: 5′-GACCATACTCCCCCCGGAACC-3′. Briefly, DNA was denatured at 94°C for 5 min and amplified by 30 cycles of denaturation at 95°C for 30 s, annealing at 58°C for 30 s, and extension at 72°C for 1 min. There was a final extension period at 72°C for 10 min prior to a 4°C hold. The PCR product was isolated using a Gel PCR Clean-Up Kit (Qiagen). For sequencing reactions, 25 ng of PCR product was used as template with 10 pmol of the above primers in separate reactions in a final volume of 12 μL. The samples were then sent to the Australian Genome Research Facility in Brisbane for sequencing. All new data has been deposited in GenBank (Table 1).

### Identification of microalgae and phylogenetic analysis

Nucleotide sequences were obtained from the NCBI database based on the BLAST results of each algae sequenced in this study. When sequences from multiple isolates of a species were available, two nucleotide sequences were chosen: (i) highest max score sequence, (ii) highest max score sequence with identified genus and species. Strains *Tetraselmis* sp. M8, *Chlorella* sp. BR2 and *Nannochloropsis* sp. BR2 were isolated by the authors and other strains were obtained from the Australian National Algae Culture Collection (ANACC), CSIRO and Queensland Sea Scallops Trading Pty Ltd (QSST), Bundaberg (Table 1). In total, 22 sequences from the NCBI database and eleven sequences from algae in this study were aligned with the MAFTT [44]. The resulting alignment was then manually inspected for quality and the end gaps trimmed. Phylogenetic analyses of the sequences was performed with PhyML 3.0 [45] using the ML method. Default settings were used, with the exception that 100 bootstraps were used in a nonparametric bootstrap analysis instead of an approximate likelihood ratio test as this is the more commonly used method in recent reports.

### Analytical methods

Measurement of nitrate and phosphate levels in the photobioreactor was performed using colorimetric assays (API, Aquarium Pharmaceuticals and Nutrafin, respectively). Growth rate, doubling time and lipid productivity were calculated as follows. The average growth rate was calculated using the equation μ = Ln(N_y_/N_x_)/(t_y_-t_x_) with Ny and Nx being the number of cells at the start (t_x_) and end (t_y_) of the growth phase (7 days). Average doubling time (T_Ave_) was calculated using the equation T = (t_y_-t_x_)/log_2_ (N_y_/N_x_) over the growth period of 7 days. The specific growth rate (μ_Max_) was calculated between the 2 days of maximum slope on the average cell density x-axis time plot [31], [46]. Lipid productivity (μg mL^−1^ day^−1^) was calculated as total lipid content (μg/mL) over the duration of the entire batch culture (laboratory cultures – 9 days, outdoor culture – 12 days).

### Microscopic analyses

After a lipid induction phase, microalgae cells were stained with 2 μg/mL Nile red (dissolved in acetone; Sigma, USA) for 15 minutes and photographed using a fluorescent Olympus BX61 microscope and an Olympus DP10 digital camera. Differential interference contrast (DIC) and epifluorescent (excitation: 510–550 nm, emission: 590 nm) images were obtained at 1000× magnification with oil immersion.

### Mid-scale outdoor cultivation

In order to evaluate the growth performance and lipid productivity of microalgae in a medium-scale outdoor setting, *Tetraselmis* sp. was selected and tested in a 1000 L outdoor photobioreactor built by The University of Queensland's Algae Biotechnology Laboratory (www.algaebiotech.org) between 20^th^ May 2011 to 1^st^ June 2011 (sunny conditions 22°C–26.5°C). An initial cell density of 1.3×10^6^/mL was cultured in SW + F/2 medium for 10 days (pH 8.8; maintained by the addition of CO_2_) followed by 2 days of nutrient starvation (nitrogen measurements were 0 mg/L on day 10). Cell counts were conducted on days 0, 2, 4, 6, 7, 10, 11 and 12 and cultures were checked to ensure that no contamination with other microalgae occurred. To facilitate comparison with laboratory protocols, growth parameters were determined within the first 7 days of culture. At day 10, 4 mL of culture was sampled for lipid analysis.

### Statistical analysis

Data for growth rates and lipid productivity was statistically analyzed by one-way analysis of variance (ANOVA) with different microalgae species as the source of variance and growth rate or lipid productivity as dependant variables. This was followed by Bonferroni's multiple comparisons test where appropriate.

## Results

### Strain collection, isolation and morphological and phylogenetic characterization of candidate microalgal biofuel strains

**Figure 1:**
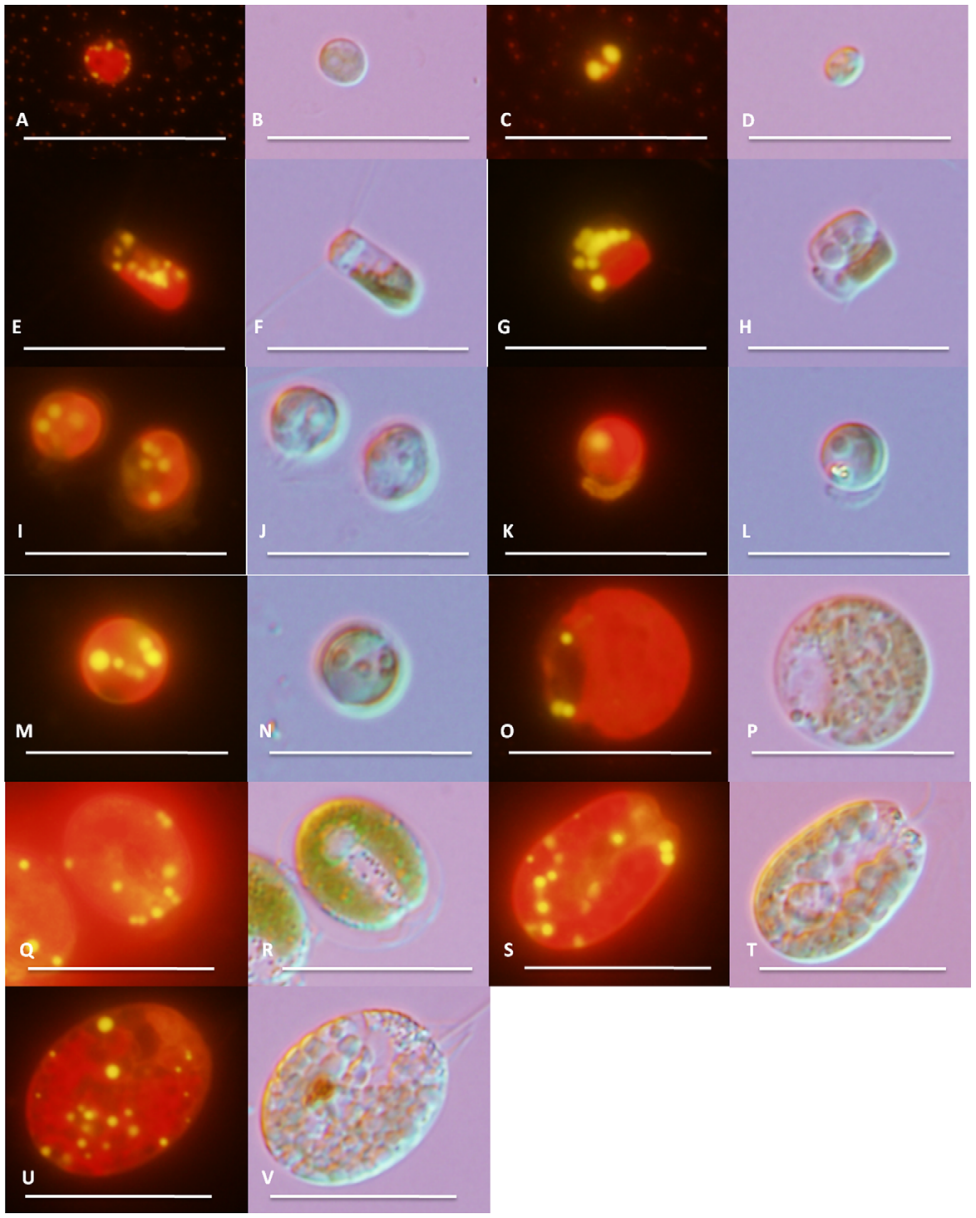
Epifluorescent (A, C, E, G, I, K, M, O, Q, S, U) and differential interference contrast (B, D, F, H, J, L, N, P, R, T, V) images of eleven microalgae used in this study. *Chlorella* sp. BR2 (A, B), *Nannochloropsis* sp. BR2 (C, D), *Chaetoceros muelleri* (E, F), *Chaetoceros calcitrans* (G, H), *Pavlova lutheri* (I, J), *Pavlova salina* (K, L), *Isochrysis* sp. (M, N), *Dunaliella salina* (O, P), *Tetraselmis chui* (Q, R), *Tetraselmis* sp. M8 (S, T) and *Tetraselmis suecica* (U, V). All images were taken at 100x magnification. Bars represent 20 µm.

Over 200 water samples were collected from diverse aquatic habitats from subtropical regions in Queensland, Australia. These included samples from rock pools in coastal areas at the Sunshine Coast, Moreton Bay, Heron Island, Gold Coast and North Stradbroke Island, as well as freshwater samples from Somerset Dam, Wivenhoe Dam and brackish samples from tidal rivers, including the Brisbane and Logan rivers. Additional microalgae strains were obtained from culture collections at ANACC, CSIRO, and two local isolates from QSST, Bundaberg. Visual microscopy (Figure 1) confirmed the isolation of uniclonal cultures. Morphological comparisons to other described microalgae suggested that these strains belonged to the genera *Tetraselmis, Chlorella, Nannochloropsis, Dunaniella, Chaetoceros, Pavlova* and *Isochrysis*.

Nile red staining and growth analysis (Table 2, Figures 1) revealed eleven candidate strains that met the criteria required for biodiesel production (i.e. easy cultivation with no special nutrient requirements, fast growth rate, seawater-strength (35 ppt) salinity tolerance and high lipid production). One promising freshwater culture (*Chlorella* sp. BR2) was also included. Under nutrient-deprived conditions, lipids produced by microalgal cells were observed as bright yellow globules when stained with Nile red and viewed under epifluorescent light (Figure 1).

**Table 2:** Growth rate analysis of eleven microalgae strains during growth phase (7 days) of batch culture.

Species	μ_Ave_	μ _Exp_	Day of μ _Exp_	DT _Ave_ [days]	Cell density_Max_ [x10^6^cells mL^−1^]	Dry weight (g L^−1^)
*Nannochloropsis* sp. BR2	0.32	0.62^c, d^	2–4	2.18^c^	48.4	0.53
*Tetraselmis* sp. M8	0.35	0.93^a, b^	2–4	2.00^c^	2.07	0.75
*T. chui*	0.35	1.03^a^	2–4	1.98^c^	1.56	0.42
*T. suecica*	0.37	0.5^d^	0–2	1.85^b, c^	1.52	0.73
*D. salina*	0.30	0.76^a, b, c, d^	2–4	2.31^c^	2.14	0.37
*C. calcitrans* 1	0.34	0.59^c, d^	0–2	2.03^c^	4.71	n/a
*C. muelleri* 1	0.35	0.71^a, b, c, d^	0–2	1.94^b, c^	4.65	0.50
*I. galbana* 1	0.35	0.61^b, c, d^	0–2	1.96^b, c^	4.45	0.45
*P. lutheri* 1	0.48^a^	0.76^a, b, c, d^	0–2	1.45^a^	3.95	0.45
*P. salina*	0.45^a^	0.88^a, b, c^	2–4	1.54^a, b^	5.47	1.68
*Chlorella* sp. BR2	0.34	0.86^a, b, c^	0–2	2.06^c^	13.8	0.59
*Tetraselmis* sp.M8^3^	0.47	0.48	6–7	1.45	1.61	0.58

1 Value represents mean of two replicate samples.

2 Different letter superscripts down a column indicate significant difference at 95% level (ANOVA, Bonferroni's test; P<0.05).

3 Mid-scale outdoor culture.

**Figure 2:**
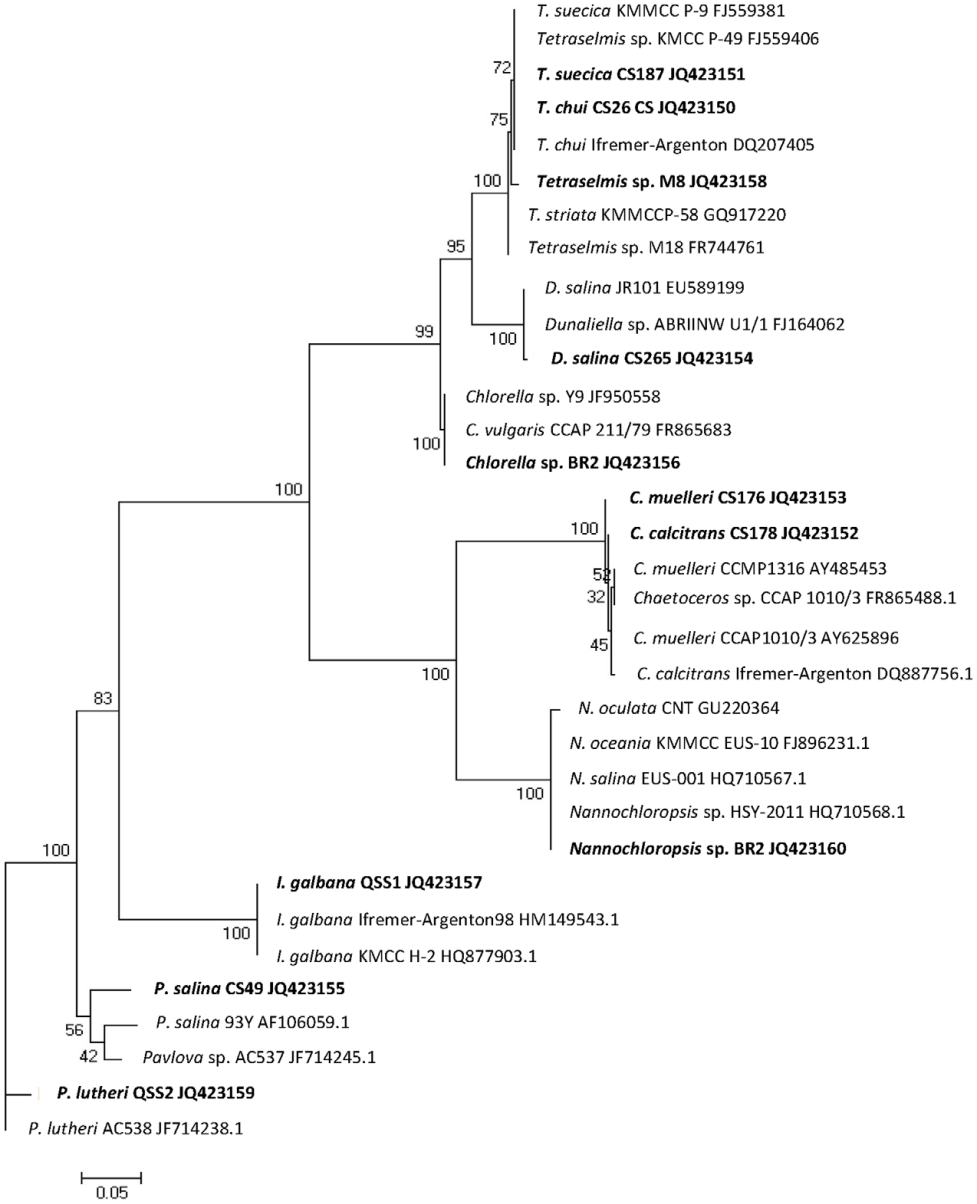
Maximum likelihood phylogenetic tree of 18S rRNA gene sequences from microalgae used in this study. Selected sequences from the NCBI database were also included (see Methods for selection criteria). Microalgae analyzed in this study are shown in bold. Numbers represent the results of 100 bootstrap replicates.

To specify the identity of the microalgae strains used in our experiments, a partial 18S region of the ribosomal RNA gene was amplified by PCR and sequenced. The obtained sequences were then compared to existing sequences in the NCBI database by the BLAST algorithm (for Genbank accession numbers see Table 1). Homology (sequence identity) searches confirmed a close relationship of the isolated candidate strains *Chlorella* sp. BR2, *Nannochloropsis* sp. BR2 and *Tetraselmis* sp. M8 with other members of the genera *Chlorella* and *Tetraselmis*. *Chlorella* sp. BR2 had a sequence identity of 99% with *Chlorella* sp. Y9, (Genbank Acc. No. JF950558) and *Chlorella vulgaris* CCAP 211/79 (Acc. No. FR865883). *Tetraselmis* sp. M8 shared a sequence identity of 99% with *Tetraselmis suecica* (CS-187) and *Tetraselmis chui* (CS-26). To characterize the diversity of the 11 microalgae strains and their relationship to other microalgae, the obtained sequences from this study were phylogenetically analyzed. The obtained maximum likelyhood phylogenetic tree (Figure 2) depicts the placement of each microalgae strain used in this study with chosen BLAST results.

BLAST 18S rRNA sequence comparison of eleven strains from this study to each other and the NCBI database (Figure 2) confirmed the taxonomic classification (suggested by microscopic studies or CSIRO/QSST) in all species based on the maximum score, while revealing high similarity within a species.

### Comparison of growth rates, doubling times and cell densities of microalgae strains

**Figure 3:**
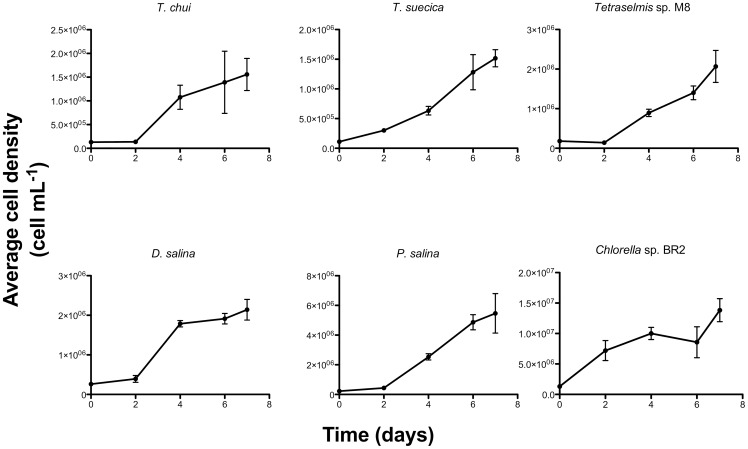
Growth curves of different microalgae in this study. *T. chui*, *T. suecica*, *Tetraselmis* sp. M8, *D. salina*, *P. salina* and *Chlorella* sp. BR2. Shown are average cell densities ± SD from three biological replicates.

**Figure 4:**
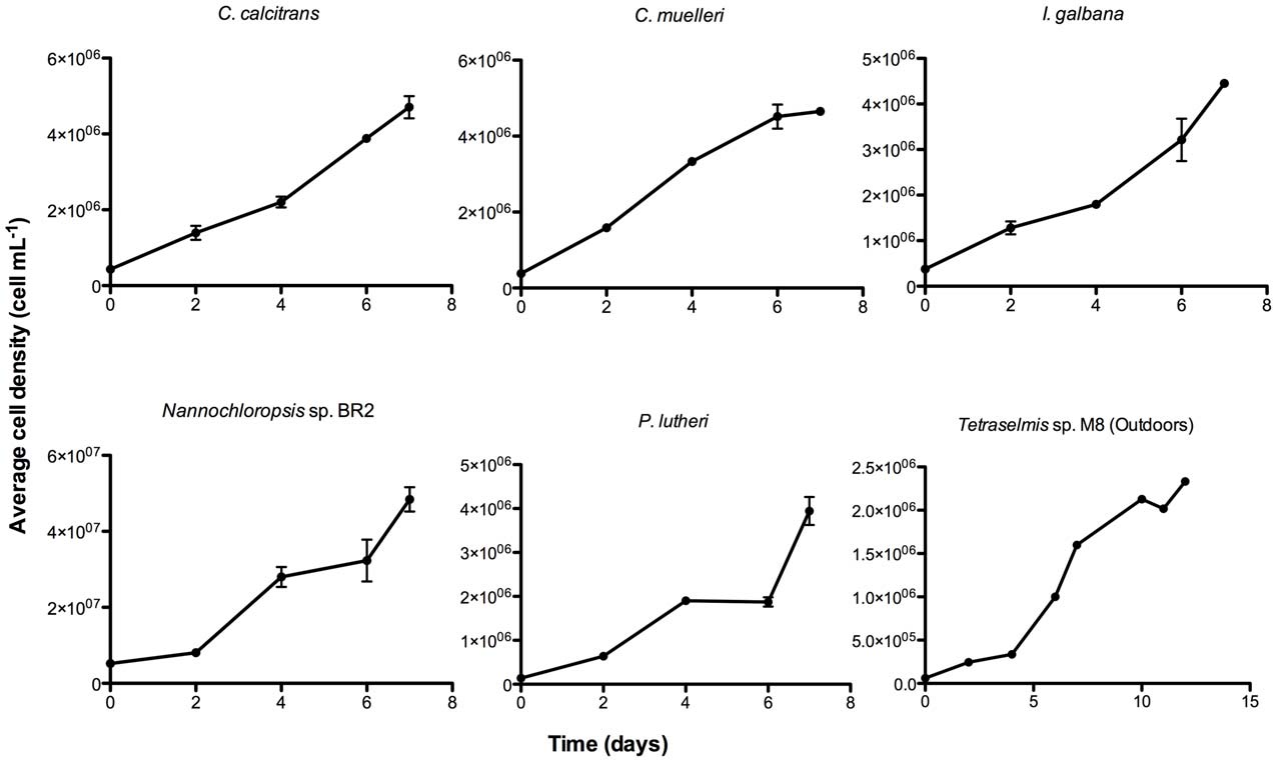
Growth curves of different microalgae in this study. *C. calcitrans*, *C. muelleri*, *I. galbana*, *Nannochloropsis* sp. BR2, *Chlorella* sp. BR2, *P. lutheri* & *Tetraselmis* sp. M8 (Outdoors). Shown are average cell densities ± SD from two biological replicates (3 replicates for *Nannochloropsis* sp. BR2 & 1 for *Tetraselmis* sp. M8 (Outdoors)).

To determine and compare growth rates, doubling times and cell densities, all microalgae strains were grown as three side-by-side cultures. After inoculation, an initial lag phase was observed in most cultures, except *Chorella* sp. BR2, *C. calcitrans*, *C. muelleri* and *I. galbana*, where exponential growth was observed immediately upon inoculation (Figures 3–4). Exponential growth in all cultures occurred till day 7 but for *D. salina*, *P. lutheri*, *Chlorella* sp. BR2 and *Nannochloropsis* sp. BR2, a lag phase was observed on day 4. *D. salina* culture remained in lag phase till day 7, while *P. lutheri*, *Chlorella* sp. BR2 and *Nannochloropsis* sp. BR2 resumed growth after day 6.

The highest average growth rate (μ_ave_) was found for *P. lutheri* (0.48 μL^−1^) and *P. salina* (0.45 μL^−1^) (Table 2), that were significantly (p<0.05) higher to all other species that had a μ_ave_ of 0.34 μL^−1^. Specific growth rates (μ_exp_), were also compared with ANOVA, revealing that *T. chui* had the highest μ_exp_ at 1.03 μL^−1^, followed by *Tetraselmis* sp. M8 (0.93 μL^−1^) and *P. salina* (0.88 μL^−1^). The fastest doubling times that were significantly different to the others were found for *P. lutheri* (1.45 days) and *Tetraselmis* sp. M8 (outdoor) (1.48 days) (Figure 3), while other microalgae strains had an average doubling time of 2.06 days. Maximum growth occurred during day 0 to day 4.

### FAME productivity and fatty acid composition

**Figure 5:**
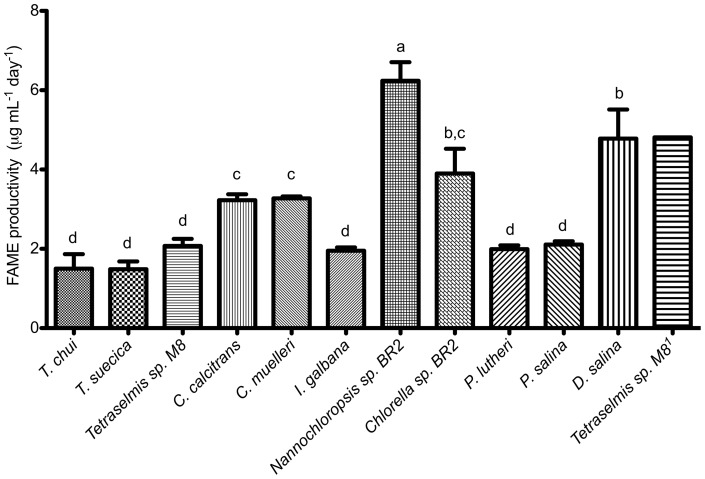
FAME levels of microalgae strains grown in batch culture (7 days growth + 2 days starvation by replacement of medium with seawater). Values shown are the averages of three biological replicates ± SD (except *Tetraselmis* sp.^1^). Different superscripts indicate significant difference at 95% level (ANOVA, Bonferroni's test; P<0.05). ^1^Mid-scale outdoors culture.

GC/MS analysis revealed *Nannochloropsis* sp. (6.24 μg mL^−1^ day^−1^) to be the highest FAME producer (ANOVA, P<0.05 in all cases), followed by *D. salina* (4.78 μg mL^−1^ day^−1^; ANOVA, P<0.05 in all cases except *Chlorella* sp. BR2, 3.9 μg mL^−1^ day^−1^) (Table 3; Figure 5). On the other hand, *T. chui* (1.5 μg mL^−1^ day^−1^) and *T. suecica* (1.49 μg mL^−1^ day^−1^) were the lowest FAME producers. The FA profile of *Nannochloropsis* sp. BR2, *C. calcitrans* and *C. muelleri* consisted predominantly of C16, C16∶1 and C20∶5 (>70% in total), while *Chaetoceros* strains produced C14 (10.5–11.6%). *Tetraselmis* sp. M8 contained most notably C18∶3 (28.9%) and C16 (22.5%), as well as C18∶2s (11.7%). *D. salina* and *Chlorella* sp. BR2's FA profile consisted mostly (nearly 90%) of C16, C18 and their unsaturated derivatives. In *T. chui* and *T. suecica*, C16 (35–37%), unsaturated C18s (37–43%) and unsaturated C20s (8–12%) were the main FAs. *I. galbana*'s FA profile was spread across C14 (19%), C16 (16%), C18∶1 (22%), C20∶3 (22%) and C20∶6 (12%). Approximately 44% of *P. salina*'s FAs consist of C14 and C16 FAs, with C20∶5 and C22∶6 FAs accounting for another 26%. *P. lutheri*'s FA profile consisted largely of C16 (25%), C16∶1 (29%), C20∶5 (22%) and C14 (11%).

**Table 3:** Fatty acid composition in percentage of total FAME of different subtropical Australian microalgae strains after batch culture (7 days growth +2 days starvation).

	*Nannochloropsis* sp.	*T.* *chui*	*T.* *suecica*	*Tetraselmis *sp. M8	*D. salina*	*C. calcitrans*	*C. muelleri*	*Isochrysis *sp.	*P. lutheri*	*P. salina*	*Chlorella *sp.	*Tetraselmis *sp. M8
Fatty acid	BR2										BR2	outdoor
C12	0.2	0.1	0.1	-	0.1	-	-	-	-	0.2	0.5	0.8
C14	3.5	0.9	0.9	0.4	0.6	10.5	11.6	19.2	11.4	19.4	0.9	4.2
C15	0.4	0.1	0.2	-	-	-	-	-	-	-	0.2	0.5
C16	33.0	37.3	35.2	22.5	24.7	23.3	26.2	16.4	25.0	24.8	30.9	20.8
C16∶1	26.8	2.5	2.3	1.1	2.9	34.1	29.7	2.0	19.1	3.6	4.4	1.3
C16∶2	0.4	-	-	5.0	2.5	1.5	2.7	0.9	3.1	-	3.4	-
C16∶3	-	0.2	-	-	2.9	4.0	5.5	-	-	-	7.8	0.1
C16∶4	-	-	-	-	11.6	-	-	-	-	-	-	-
C17	0.4	0.1	-	4.5	-	1.6	1.8	-	-	-	0.4	2.5
C18	3.0	9.0	8.8	3.0	5.8	5.1	4.5	4.4	4.8	8.3	9.7	10.1
C18∶1	6.0	13.8	15.3	9.1	5.6	5.8	1.7	21.7	1.3	2.0	9.2	13.6
C18∶2	0.9	8.8	19.7	11.7	7.6	0.1	0.2	0.7	-	1.1	7.9	7.0
C18∶3	0.4	15.1	8.8	28.9	33.8	0.0	0.4	3.1	0.1	1.3	22.8	11.1
C18∶4	-	-	-	-	-	-	-	-	-	6.1	-	12.7
C20	0.2	0.5	0.5	-	0.1	-	-	-	-	0.4	0.9	-
C20∶1	-	1.8	2.1	-	0.1	-	-	5.9	0.1	-	0.8	4.6
C20∶4	5.9	2.6	3.3	3.4	-	0.9	1.4	13.9	6.1	-	0.1	0.1
C20∶5	18.8	7.2	2.9	10.6	1.2	12.7	14.0	0.0	21.8	16.1	-	10.6
C22	-	-	-	-	-	-	-	-	-	-	-	-
C22∶4	-	-	-	-	-	-	-	-	-	6.3	-	-
C22∶6	-	-	-	-	0.4	0.3	0.4	11.8	7.3	10.5	-	-
**Total saturated (%)**	40.7	47.9	45.6	30.4	31.4	40.5	44.0	39.9	41.1	53.0	43.6	38.9
**Total monounsaturated (%)**	32.8	18.2	19.7	10.2	8.6	40.0	31.4	29.6	20.5	5.5	14.4	19.5
**Total polyunsaturated (%)**	26.5	34.0	34.7	59.5	60.0	19.5	24.6	30.5	38.3	41.4	42.0	41.7
**Total FAMEs (μg mL^-1^)**	56.1	13.5	13.4	18.7	43.0	29.0	29.5	17.6	17.9	19.0	31.4	57.7
**Total FAME/dry weight (%)**	10.6	3.2	10.8	2.5	11.4	-	5.9	3.9	4.0	1.2	5.3	9.9

On average, saturated FAs accounted for 40% of the total FAs in this study, consisting mostly of C16 (27.2%), C14 (7.2%) and C18 (6%). Similar amounts (37.4%) of FAs were polyunsaturated and included EPA C20∶5 (9.6%), ALA C18∶3 (10.4%) and DHA C22∶6 (3.9%). Monounsaturated FAs accounted for 21% of the total FAs, consisting mostly of C16∶1 (11.7%) and C18∶1 (8.3%). *P. salina* was found to have the highest saturated FA (53%), *C. calcitrans* the highest monounsaturated FA (40%), and *D*. salina the highest polyunsaturated FA content (60%). C16 was found to be a major FA (17–37%) in all the strains tested, particularly in *T*. *chui*, *T. suecica* and *Nannochloropsis* sp. BR2. C16∶1 FAs were predominantly found in *C. calcitrans*, *C. muelleri* and *Nannochloropsis* sp. BR2, while highest C14 content was found in *P. salina* and *I. galbana*. *I. galbana* also had the highest content of C18∶1 FAs, while C18∶3 FAs were predominantly found in *D. salina*, *Chlorella* sp. BR2 and *Tetraselmis* sp. M8. *Nannochloropsis* sp. BR2 and *P. lutheri* both had the highest content of EPA C20∶5 FAs while DHA C22∶6 was predominantly found in *P. salina*. *D. salina* was the only strain found to produce C16∶4. It should be noted that due to the small culture volumes in this study certain fatty acids may have remained undetectable.

### Outdoor scale-up

The highest lipid productivity for the microalgae strains tested in this study, was measured for *Nannochloropsis* sp. BR2 (Figure 5). However, based on its versatility and resourcefulness of fatty acids, its short doubling times, its ease of handling, and its potentially better lipid extraction efficiency, *Tetraselmis* sp. M8 was identified as a suitable candidate for large-scale cultivation whose FAME profiles would also meet the criteria for a future microalgae biorefinery. To compare laboratory cultivation with larger outdoor cultivation, *Tetraselmis* sp. M8 culture was grown in a 1000 L closed photobioreactor that was inoculated with 20 L of saturated culture. This mid-scale outdoor culture achieved a cell density of 1.6×10^6^ cells mL^−1^ on day 7, eventually arriving at 2.3×10^6^ cells mL^−1^ on day 10. Maximum growth rate was found between day 4 and 6 (Table 2) and was similar to average growth rates (0.47 μL^−1^ and 0.5 μL^−1^, respectively). The culture entered stationary phase during starvation (after day 10), and cell count did not increase. The mid-scale, outdoor cultivation of *Tetraselmis* sp. M8 achieved a FAME productivity of 4.8 μL mL^−1^ day^−1^, consisting mostly of C16 (20.8%), C18 (10.1%) and C18 unsaturated fatty acids (44.6%).

## Discussion

In a microalgae-based oil industry, high oil productivity is crucial to achieving commercial feasibility. While growth conditions (e.g. solar radiation and temperature) and culture management are important, the suitable microorganism is fundamental to produce the desired quality and quantity of oil. A suitable microalgae strain must have high lipid productivity, either by possessing a high basal lipid content and/or be inducible to accumulate significant amounts of lipids. The selected strain should also be easily harvested, amenable to efficient oil extraction and flexible enough to adapt to changing physio-chemical conditions in an outdoor environment [11]. Thus, a locally isolated strain would likely adapt better to local changing environmental conditions and provide a more stable and productive culture.

Sampling at local waterways focused on inter-tidal rock pools, where the microclimate alters frequently between optimal growth conditions and unfavorable conditions (e.g. low nutrients, micro-oxic conditions, anaerobiosis, low/high light or dry, hot or cold conditions or rapid changes in salinity). Sampling at such locations was considered advantageous because suboptimal conditions would require the algae there to accumulate photo-assimilates such as starch or lipids that have important storage functions in order to survive, thereby increasing the chances of obtaining high lipid content strains [3]. This was followed by an isolation process targeted to select for high growth rate microalgae strains that could be induced to accumulate lipids under nutrient-deprived conditions. Isolation of uni-clonal microalgae strains by serial dilution and plating in F-supplemented medium was designed to select strains which grew well in F/2 medium, a common nutrient mix used for microalgae culture [31], [32], [40], [41]. Serial dilutions would also select for fast growing strains, which would inevitably dominate a culture. Special attention must be given to ensure that a single fast growing strain does not dominate other potentially high lipid content strains but that may have a slower growth rate. After 48 hours of nutrient deprivation, Nile red staining of the isolated uni-clonal cultures revealed several strains with substantial lipid producing potential. An inherent problem with using Nile red staining was that differences in cell wall structure between species do not allow for equal staining and prevented accurate comparison of lipid productivity between species. For this reason some species with thick cell walls (e.g. some other *Nannochloropsis* species) that were not included in the subsequent analysis may still have a strong potential as future microalgae crops.

A standard protocol was established to identify the top FAME-producing microalgae strains by comparing the growth rates, FAME productivity and composition of the 11 microalgae strains in this study. Growth rate and FAME productivity data was then compared with other literature (Table 4). It is crucial that any comparison must take into consideration the different growth conditions, culture system and lipid analysis methods (available in Table S1). Both average growth rate (μ_ave_) and specific growth rate (μ_exp_) of the 11 analyzed microalgae strains were calculated from cell count growth curves (Figures 3–4). Overall, μ_ave_ found in the present study were similar or higher than μ_ave_ published by [36] and [34], aside from [32] which had nearly twice the μ_ave_ (Table 4)_._ The specific growth rate (μ_exp_) of microalgae is more widely reported in the literature, although many studies only present growth in biomass productivity [11], [30], [33], [35], [47]. Comparison with available literature revealed the present study's overall μ_exp_ to be higher than most, with the exception of microalgae from three publications [40], [48], [49]. The overall high growth rates of this study were observed despite a lack of culture conditions such as air bubbling, CO_2_ supplementation and longer photoperiods available in other studies (Table 4; Supplementary Table S1). This could be a result of the increased nutrient availability from the F media in comparison with other studies that utilize F/2 media [31], [34], [36]. Increase in nutrient availability, particularly nitrogen has been documented to increase growth rate [29], [30], [50], particularly when the nitrogen source in F/2 media, KNO_3_ is low (0.75 mM). A previous study on *Nannochloropsis* discovered light intensity to only have a slight effect on growth rates [47], especially during low cell densities (Skye Thomas-Hall, personal communication) and growth rate discrepancies may be due to differences in prior culture history [51]. Ultimately, *T. chui* and *Tetraselmis* sp. M8 were found to have the highest μ_exp_. *Tetraselmis* strains were also the fastest growers in two other studies, [31] and [34]. The growth rate of *Nannochloropsis* sp. in this study was below average, contrary to findings by Huerlimann et al. [31]. FAME analysis by GC/MS revealed *Nannochloropsis* sp. BR2 to be the highest TAG producer, followed by *D. salina* and *Chlorella* sp. BR2. These three strains have been found to also be high lipid producers in other studies. Rodolfi et al. [11] compared the lipid productivity of 30 microalgae strains and found *Nannochloropsis oculata* and *Chlorella* amongst the best producers of lipids, both indoors and outdoors. Likewise, Huerlimann et al. [31] investigated the lipid content of five tropical microalgae and discovered *Nannochloropsis* sp. to be the highest lipid producer. A strain of *Chlorella* was similarly found to be a high lipid producer in an evaluation of ten microalgae strains for oil production [33]. Surprisingly, *Isochrysis* sp., a high lipid producing strain in other studies, [34] and [35], was found to have one of the lowest lipid production rates in this study. Likewise, *Tetraselmis* strains, top lipid producers in other studies, [31] and [11], produced the least amounts of lipids in this study.

**Table 4:** Comparison of FAME productivity (μg mL^−1^ day^−^1) of present study microalgae with lipid productivity of microalgae species from other references.

Species	Lipid productivity [μg mL^−1^ day^−1^]	References
***Nannochloropsis*** ** sp. BR2**	6.2	This studyGCMS, AG
*Nannochloropsis* sp.	4.6	Huerlimann et al. (2010)12h
*Nannochloropsis* sp.	48.0	Rodolfi et al. (2009)24h, CO2
*Nannochloropsis* sp.	37.6	Rodolfi et al. (2009)24h, CO2
*Nannochloropsis* sp.	60.9	Rodolfi et al. (2009)24h, CO2
*Nannochloropsis oculata*	10.0	Converti et al. (2009)24h, CO2
***Tetraselmis*** ** sp. M8**	2.1	This studyGCMS, AG
***Tetraselmis*** ** sp. M8 (outdoor)**	4.8	This studyGCMS
*Tetraselmis* sp.	18.6	Huerlimann et al. (2010)12h
*Tetraselmis* sp.	43.4	Rodolfi et al. (2009)24h, C02
*Tetraselmis* sp.	10.7	Patil et al. (2007)GCMS, 24h, CO2
***Tetraselmis chui***	1.5	This studyGCMS, AG
*Tetraselmis chui*	27.0	Rodolfi et al. (2009)24h, CO2
***Tetraselmis suecica***	1.5	This studyGCMS, AG
*Tetraselmis suecica*	36.4	Rodolfi et al. (2009)24h, CO2
***Dunaliella salina***	4.8	This studyGCMS, AG
*Dunaliella salina*	33.5	Takagi et al. (2006)
***Chaetoceros muelleri***	3.3	This studyGCMS, AG
*Chaetoceros muelleri*	21.8	Rodolfi et al. (2009)24h, CO2
***Chaetoceros calcitrans***	3.2	This studyGCMS, AG
*Chaetoceros calcitrans*	17.6	Rodolfi et al. (2009)24h, CO2
*Chaetoceros* sp.	16.8	Renaud et al. (2002)* 12h
***Isochrysis galbana***	2.0	This studyGCMS, AG
*Isochrysis* sp.	24.9	Renaud et al. (2002)* 12h
*Isochrysis* sp.	12.7	Huerlimann et al. (2010)12h
*Isochrysis* sp.	37.7	Rodolfi et al. (2009)24h, CO2
*I. galbana*	12.4	Patil et al. (2007)GCMS, 24h, CO2
***Pavlova lutheri***	2.0	This studyGCMS, AG
*Pavlova lutheri*	50.2	Rodolfi et al. (2009)24h, CO2
***Pavlova salina***	2.1	This studyGCMS, AG
*Pavlova salina*	49.4	Rodolfi et al. (2009)24h, CO2
*Pavlova* sp.	21.7	Patil et al. (2007)GCMS, 24h, CO2
***Chlorella*** ** sp.**	3.9	This studyGCMS, AG
*Chlorella* sp.	7.1	Chen et al. (2010)^AG^
*Chlorella* sp.	20.0	Converti et al. (2009)24h, CO2
*Chlorella* sp.	42.1	Rondolfi et al. (2009)24h, CO2
*Chlorella sorokiana*	44.7	Rondolfi et al. (2009)24h, CO2
*Chlorella sorokiana*	1.0	Illman et al. (2000)24h, CO2
*Chlorella vulgaris*	5.3	Illman et al. (2000)24h, CO2

* Calculated total lipid content (μg mL^−1^).

GCMS Values obtained by GC/MS.

24h Cultures grown with 24 h light and air.

12h Cultures grown with 12h light and air.

CO2 Cultures grown with air supplemented with CO_2._

AG Cultures grown with agitation.

For a full comparison of culturing conditions see Table S1.

Variations in species strains, growth conditions, experimental design and lipid extraction/analysis methods make quantitative comparisons of lipid productivity and FA content between studies very difficult (Supplementary Table S1). Nevertheless, when compared with Patil et al [35], who similarly analyzed FAME productivity by GC/MS, the total FAME/dry weight (%) of *Nannochloropsis* sp. BR2 and *Tetraselmis* sp. M8 was found to be higher, while *I. galbana* produced the same amount of FAME/dry weight. However, GC/MS obtained FAME productivity of this study was found to be lower than other sources (except for [37])(Table 4) that utilized solvent and gravimetric methods to measure total lipids. This was expected as solvent and gravimetric methods would include FFAs, TAGs and other lipid classes such as polar lipids (e.g. phospholipids and glycolipids) [6], wax esters [52], isoprenoid-type lipids, [53], sterols, hydrocarbons and pigments. Furthermore, different growth conditions in other studies such as growth enrichment with carbon dioxide [48], [54], increased photoperiods and light intensity [55], different media volumes and larger initial inoculum would explain for the increased lipid productivity in other studies. This is most evident in the study by Rodolfi et al. [11], where similar strains of *P. salina* CS-49 and *C. calcitrans* CS-178 were studied under different conditions to reveal significantly different results. It should be noted that the conditions of the current experimental design were not meant to achieve maximum lipid production but to determine the best lipid producing candidates under standard “unoptimized lab conditions”, which were *Nannochloropsis* sp. BR2, *D. salina* and *Chlorella* sp. BR2. Higher confidence in the data may be obtained by growing cultures completely independently (i.e. experiments carried out separately at different times with a different culture). Subsequent studies may focus on the comparison of best strains under fully optimized and/or large-scale commercial conditions. In our study, *Tetraselmis* sp M8 was chosen for a scale-up study based on its fast growth rates, culture dominance and ease of harvesting by settling. A comparison of the indoor laboratory conditions to mid-scale (1000 L) outdoor conditions showed that lipid productivity more than doubled under these conditions. Although further long-term studies will be required, these preliminary findings demonstrate the potential for optimization and emphasize that outdoor and large-scale conditions differ strongly from laboratory conditions.

Suitable candidates for biodiesel production require not only high lipid productivity, but also suitable FA content. Recommended FAs for good biodiesel properties include C14∶0, C16∶0, C16∶1, C18∶0, C18∶1, C18∶2 and C18∶3 [3], [56]. In this study, analyses of FA profiles revealed *Nannochloropsis* sp. BR2, *Chlorella* sp. BR2 and *Chaetoceros* strains (*C. calcitrans* and *C. muelleri*) to be the best candidates (Table 3). In addition to having the highest lipid productivity, the recommended FAs for biodiesel accounted for 73.6% of the total FAs in *Nannochloropsis* sp. BR2, in particular C16 (33%) and C16∶1 (26.8%). Huerlimann et al. [31] reported a similar FA composition of *Nannochloropsis* sp. following nutrient deprivation, while Patil et al. [35] also reported *Nannochloropsis* sp. to have the highest C16 and C16∶1 content. *Chlorella* sp. BR2 presented slightly lower lipid productivity although having more desired FAs for biodiesel (81.4%). It also had a higher C18 (9.7%) and unsaturated C18 content (39.9%) if compared to *Nannochloropsis* sp. BR2 or the *Chaetoceros* strains; making it more desirable for the production of biodiesel with a higher cold filter plugging point (CFFP) for better performance at low temperatures [3]. Both *C. calcitrans* and *C. muelleri* are good candidates despite only having mediocre lipid productivity due to high levels of C14 FAs (10.5% and 11.6% respectively) and recommended FAs for biodiesel (78.9% and 74.5% respectively). The FA content of *C. calcitrans* was observed in accordance to Lee et al. [34] during low nitrogen conditions, which caused an increase in saturated FAs like C16. *D. salina* was not considered a suitable candidate for biodiesel despite its high lipid productivity due to high levels of PUFAs (C16∶4 – 11.6%. C18∶3 – 33.8%). Low levels of PUFAs, as evident in *Nannochloropsis* sp. and *C. calcitrans* are desired for biodiesel production as it reduces the need for treatments such as catalytic hydrogenation. *Nannochloropsis* sp. BR2, *C. calcitrans* and *C. muelleri* also exhibited C20∶5 (EPA) (18.8%, 12.7% and 14% respectively) that would allow for a biorefinery approach to biodiesel production. It should be noted, however, that microalgal biodiesel is likely to be first used as a drop-in fuel in the future which would allow to achieve blends with the desired fuel properties from most microalgae species.

Commercially feasible production of microalgal biodiesel would require a biorefinery approach to produce biodiesel as well as other value-added products such ω-3 FAs and protein-rich biomass. Microalgae possess the potential to produce high amounts of ω-3 FAs such as EPA (C20∶5) and DHA (C22∶6) that are used as dietary supplements. The best candidates for EPA and DHA production in this study were found to be *Nannochloropsis* sp. BR2 and the *Pavlova* strains (*P. salina* and *P. lutheri*). Overall, *Nannochloropsis* sp. BR2 produced the highest amounts of ω-3 FAs on account of its high overall lipid and EPA content (18.8%). *P. lutheri* exhibited the highest proportional content of EPA (21.8%), while *Isochrysis* sp. had the highest DHA content (11.8%). The ω-3 FA contents of *Nannochloropsis* sp. and the *Pavlova* strains were comparable to previously published values [31], [35], [57].

The use of a nutrient starvation phase to improve TAG productivity (particular C16∶0 and C16∶1) for biodiesel production was successful as C16 and C16∶1 FAs were found to be the predominant FAs in the present study. During nutrient limiting conditions, unsaturated FAs are consumed as an energy source and saturated FAs are accumulated [58]. The increase of the % of saturated and monounsaturated FAs during starvation have been well documented in literature for several other species [34], [59], [60]. While this may prove useful for biodiesel production, the reduction in PUFAs is a problem for ω-3 FA production that has been documented [31], [34]. Nevertheless, EPA and DHA contents have been reported to remain consistent despite changes in nutrient level for *T. tetrathele* [40], which may explain the high levels of PUFA observed in *Tetraselmis* sp.

In a 1000 L-outdoor setting, *Tetraselmis* sp. M8 was found to have an increased μ_Ave_ despite a longer lag phase. Cell density achieved by outdoor grown *Tetraselmis* sp. M8 was similar to other large-scale cultures of *Tetraselmis* [61]. FAME productivity and composition were also analyzed, which revealed a near tripling of FAME productivity as well as altered FA composition. High amounts of C16∶2, C18∶2, C18∶3 previously detected in laboratory-grown *Tetraselmis* sp. M8 was found reduced, while higher amounts of recommended FA for biodiesel (particularly C14, C18 & C18∶1) were present. The increase in FAME productivity and desirable FA composition of *Tetraselmis* sp. M8 in a mid-scale setting demonstrates that the microalgae isolation and selection technique used in this study can lead to the identification of microalgae strains with potential for large-scale cultivation. Additional factors to be considered for large-scale production include harvesting and oil extraction properties of different microalgae. For example, we noticed that our *Tetraselmis* strains may lose their flagella during stress conditions, resulting in rapid settling that allows easy harvesting/dewatering. Small microalgae, such as *Nannochloropsis* sp., on the other hand may instead be harvested by froth flotation or other techniques, but our results indicate that Nile red staining and lipid extraction may be compromised by thick cell walls in this strain.

## Supporting Information

Table S1 Comparison of FAME productivity (μg mL^−1^ day^−^1) of present study microalgae with lipid productivity of microalgae species from other references (including a full comparison of culturing conditions).
(PDF)

## References

[1] Chisti Y (2007). Biodiesel from microalgae.. Biotechnol Adv.

[2] Malcata FX (2011). Microalgae and biofuels: A promising partnership?. Trends Biotechnol.

[3] Schenk PM, Thomas-Hall SR, Stephens E, Marx UC, Mussgnug JH (2008). Second generation biofuels: high-efficiency microalgae for biodiesel production.. Bioenerg Res.

[4] Dermirbas A (2009). Potential resources of non-edible oils for biodiesel.. Energy Sources Part B – Economics Planning & Policy.

[5] Durret T, Benning C, Ohlrogge J (2008). Plant triacylglycerols as feedstocks for the production of biofuels.. Plant J.

[6] Hu Q, Sommerfeld M, Jarvis E, Ghirardi M, Posewitz M (2008). Microalgal triacylglycerols as feedstocks for biofuel production: perspectives and advances.. Plant J.

[7] Ahmad AL, Mat Yasin NH, Derek CJC, Lim JK (2011). Microalgae as a sustainable energy source for biodiesel production: A review.. Renew Sustain Energ Rev.

[8] Chisti Y (2008). Biodiesel from microalgae beats bioethanol.. Trends Biotechnol.

[9] Gouveia L, Oliveria A (2009). Microalgae as a raw material for biofuels production.. J Ind Microbiol Biotechnol.

[10] Kliphus AMJ, Lenneke DW, Vejrazka C (2010). Photosynthetic Efficiency of *Chlorella sorokiana* in a turbulently mixed short light-path photobioreactor.. Biotechnol Progr.

[11] Rodolfi L, Zittelli GC, Bassi N, Padovani G, Biondi N (2009). Microalgae for oil: strain selection, induction of lipid synthesis and outdoor mass cultivation in a low-cost photobioreactor.. Biotechnol Bioeng.

[12] Zeiler KG, Heacox DA, Toon ST, Kadam KL, Brown LM (1995). The use of microalgae for assimilation and utlization of carbon dioxide from fossil fuel-fired power plant flue gas.. Energ Convers Manag.

[13] Wang B, Li YQ, Wu N, Lan CQ (2008). CO_2_ bio-mitigation using microalgae.. Appl Microbiol Biotechnol.

[14] Bridgewater A, Maniatis K, Archer M, Barber J (2004). The production of biofuels by thermal chemical processing of biomass..

[15] Pulz O, Gross W (2004). Valuable products from biotechnology of microalgae.. Appl Microbiol Biotechnol.

[16] Ruxton CHS, Reed SC, Simpson MJA, Millington KJ (2004). The health benefits of omega-3 polyunsaturated fatty acids: a review of the evidence.. J Human Nutrition Dietetics.

[17] Cavalli RO, Lavens P, Sorgeloos P (1999). Performance of *Macrobrachium rosenbergii* broodstock fed diets with different fatty acid composition.. Aquaculture.

[18] Doroudi MS, Southgate PC, Mayer RJ (1999). Growth and survival of blacklip pearl oyster larvae fed different densities of microalgae.. Aquaculture Internat.

[19] Emata AC, Ogata HY, Garibay ES, Furuita H (2003). Advanced broodstock diets for the mangrove red snapper and a potential importance of arachidonic acid in eggs and fry.. Fish Physiol Biochem.

[20] Amaro HM, Guedes AC, Malcata FX (2011). Advances and perspectives in using microalgae to produce biodiesel.. Appl Energy.

[21] Weyer KM, Bush DR, Darzins A (2010). Theoretical maximum algal oil production.. Bioenerg Res.

[22] Dermirbas A, Dermirbas MF (2011). Importance of algae oil as a source of biodiesel.. Energy Convers Manag.

[23] Mata TM, Martins AA, Caetano NS (2010). Microalgae for biodiesel production and other applications: A review.. Renew Sustain Energ Rev.

[24] Knothe G, Gerpen JV, Krahl J (2005). The biodiesel handbook.. Urbana, IL: AOCS Press.

[25] Knothe G (2005). Dependence of biodiesel fuel properties on the structure of fatty acid alkyl esters.. Fuel Process Technol.

[26] Abu-Rezq T, Al-Musallam L, Al-Shimmari J (1999). Optimum production conditions for different high-quality marine algae.. Hydrobiologia.

[27] Chiu SY, Kao CY, Tsai MT, Ong SC, Chen CH (2009). Lipid accumulation and CO_2_ utilization of *Nannochloropsis oculata* in response to CO_2_ aeration.. Bioresour Technol.

[28] Cho S, Ji SC, Hur S, Bae J, Park IS (2007). Optimum temperature and salinity conditions for growth of green algae *Chlorella ellipsoidea* and *Nannochloris oculata*.. Fish Science.

[29] Li YQ, Horsman M, Wang B, Wu N, Lan CQ (2008). Effects of nitrogen sources on cell growth and lipid accumulation of green algae *Neochloris oleoabundans*.. Appl Microbiol Biotechnol.

[30] Chen M, Tang H, Ma H, Holland TC, Ng KYS (2011). Effects of nutrient on growth and lipid accumulation in the green algae *Dunaliella tertiolecta*.. Bioresour Technol.

[31] Huerlimann R, de Nys R, Heimann K (2010). Growth, lipid content, productivity, and fatty acid composition of tropical microalgae for scale-up production.. Biotechnol Bioeng.

[32] Renaud SM, Thinh LV, Lambrinidis G, Parry DL (2002). Effect of temperature on growth, chemical composition and fatty acid composition of tropical Australian microalgae grown in batch cultures.. Aquaculture.

[33] Araujo GS, Matos LJBL, Goncalves LRB, Fernandes FAN, Farias WRL (2011). Bioprospecting for oil producing microalgal strains: Evaluation of oil and biomass production for ten microalgal strains.. Bioresour Technol.

[34] Lee S, Go S, Jeong G, Kim S (2011). Oil production from five marine microalgae for the production of biodiesel.. Biotechnol Bioprocess Eng.

[35] Patil V, Kallqvist T, Olsen E, Vogt G, Gislerod HR (2007). Fatty acid composition of 12 microalgae for possible use in aquaculture feed.. Aquaculture Internat.

[36] Converti A, Casazza AA, Ortiz EY, Perego P, Del Borghi M (2009). Effects of temperature and nitrogen concentration on the growth and lipid content of *Nannochloropsis oculata* and *Chlorella vulgaris*.. Chem Eng Process.

[37] Illman AM, Scragg AH, Shales SW (2000). Increase in *Chlorella* strains calorific values when grown in low nitrogen medium.. Enzyme Microb Technol.

[38] Borowitzka MA (1988). Fats, oils and hydrocarbons.. Borowitzka, MA and LJ Borowitzka (Ed) Micro-Algal Biotechnology X+477p Cambridge University Press: New York, New York, USA; Cambridge, England, UK Illus.

[39] Roessler PG (1990). Environmental control of glycerolipid metabolism in microalgae – commercial implications and future-research directions.. J Phycol.

[40] de la Pena MR, Villegas CT (2005). Cell growth, effect of filtrate and nutritive value of the tropical prasinophyte *Tetraselmis tetrathele* (Butcher) at different phases of culture.. Aquaculture Res.

[41] Guillard RR, Ryther JH (1962). Studies of marine planktonic diatoms: I. *Cyclotella nana* (Hustedt) and *Detonula confervacea* (Cleve) Gran.. Canad J Microbiol.

[42] Lorenz M, Friedl T, Day JG, Andersen RA (2005). Perpetual maintenance of actively metabolizing microalgal cultures..

[43] Chomczynski P, Sacchi N (1987). Single-step method of RNA isolation by acid guanidium thiocyanate phenol chloroform extraction.. Analyt Biochem.

[44] Katoh K, Asimenos G, Toh H (2009). Multiple alignment of DNA sequences with MAFFT.. Meth Mol Biol.

[45] Guidon S, Gascuel O (2003). A simple, fast and accurate algorithm to estimate large phylogenies by maximum likelihood.. Systems Biol.

[46] Wood AM, Everroad RC, Wingard LM, A AR (2005). Chapter 18: Measuring growth rates in microalgal cluures..

[47] Pal D, Khozin-Goldberg I, Cohin Z, Boussiba S (2011). The effect of light, salinity and nitrogen availability on lipid production by *Nannochloropsis* sp.. Appl Microbiol Biotechnol.

[48] Araujo SC, Garcia VMT (2005). Growth and biochemical composition of the diatom *Chaetoceros* cf. *wighamii* Brightwell under different temperature, salinity and carbon dioxide levels. I. Protein, carbohydrates and lipids.. Aquaculture.

[49] Emdadi D, Berland B (1989). Variation in lipid class composition during batch growth of *Nannochloropsis salina* and *Pavlova lutheri*.. Marine Chem.

[50] Rocha JMS, Garcia JEC, Henriques MHF (2003). Growth aspects of the marine microalga *Nannochloropsis gaditana*.. Biomol Eng.

[51] Miyamoto K, Wable O, Benemann JR (1988). Vertical tubular reactor for microalgae cultivation.. Biotechnol Lett.

[52] Alonzo F, Mayzaud P (1999). Spectrofluorometric quantification of neutral and polar lipids in zooplankton using Nile red.. Marine Chem.

[53] Gong Y, Jiang M (2011). Biodiesel production with microalgae as feedstock: from strains to biodiesel.. Biotechnol Lett.

[54] Hu H, Gao K (2003). Optimisation of growth and fatty acid composition of a unicellular marine picoplankton, *Nannochloropsis* sp., with enrichment carbon sources.. Biotechnol Lett.

[55] Chen CH, Yeh K, Su H, Lo Y, Chen W (2010). Strategies to enhance cell growth and achieve high-level oil production of a *Chlorella vulgaris* isolate.. Biotechnol Prog.

[56] Knothe G (2008). “Designer” biodiesel: optimising fatty ester composition to improve fuel properties.. Energy Fuels.

[57] Reitan KI, Rainuzzo JR, Olsen Y (1994). Effect of nutrient limitation on fatty acid and lipid content of marine microalgae.. J Phycol.

[58] Jeh EJ, Song SK, Seo JW, Hur BK (2007). Variation in the lipid class and fatty acid composition of *Thraustochytrium aureum* ATCC 34304.. Korean J Biotechnol Bioeng.

[59] Dunstan GH, Volkman JK, Barret SM, Garland CD (1993). Changes in the lipid composition and maximization of the polyunsaturated fatty acid content of three microalgae grown in mass culture.. J Appl Phycol.

[60] Shamsudin L (1992). Lipid and fatty acid composition of microalgae used in Malaysian aquaculture as live food for the early stage of penaeid larvae.. J Appl Phycol.

[61] Okauchi M, Kawamura K (1997). Optimum medium for large-scale culture of *Tetraselmis tetrathele*.. Hydrobiologia.

