# Supplementary material for: Isolation and Evaluation of Oil-Producing Microalgae from Subtropical Coastal and Brackish Waters
Source: PLoS One. 2012 Jul 11;7(7):e40751. doi: 10.1371/journal.pone.0040751 (PMC3394722; doi:10.1371/journal.pone.0040751)
Supplement: Table S1 — Comparison of FAME productivity (μg mL−1 day−1) of present study microalgae with lipid productivity of microalgae species from other references (including a full comparison of culturing conditions). (PDF) [file pone.0040751.s001.pdf]

Supplementary Table S1

Comparison of FAME productivity (μg mL^{-1} day^{-}1) of present study microalgae with lipid productivity of microalgae species from other references (including a full comparison of culturing conditions)

Species	Growth rate μ_{Ave}	μ_{Exp}	Lipid productivity μg mL^{-1} day^{-1}	References	Air supply	Light Cycle	Light Intensity	Volume	Media
Nannochloropsis sp. E	0.32	0.6	6.2	This study[GCMS, AG]	Agitation	12L:12D	120 μmol photons m^{-2} s^{-1}	20 mL	F
Nannochloropsis sp.	-	0.3	4.6	Huerlimann et al. (2010)[12h]	Air	12L:12D	250 μmol photons m^{-2} s^{-1}	10 L	F/2
Nannochloropsis sp.	-	-	48.2	Rodolfi et al. (2009)[24h, CO2]	Air/CO_2 (95/5)	24L:0D	100 μmol photons m^{-2} s^{-1}	100 mL	F
Nannochloropsis sp.			37.6	Rodolfi et al. (2009)[24h, CO2]	Air/CO_2 (95/5)	24L:0D	100 μmol photons m^{-2} s^{-1}	100 mL	F
Nannochloropsis sp.			60.9	Rodolfi et al. (2009)[24h, CO2]	Air/CO_2 (95/5)	24L:0D	100 μmol photons m^{-2} s^{-1}	100 mL	F
N. occulata	0.07	-	10.0	Converti et al. (2009)[24h, CO2]	Inc: Air & CO_2	24L:0D	70 μE m^{-2} s^{-1}	2 L	F/2
Tetraselmis sp. M8	0.35	0.9	2.1	This study[GCMS, AG]	Agitation	12L:12D	120 μmol photons m^{-2} s^{-1}	20 mL	F
Tetraselmis sp. M8	0.47	0.5	4.8	This study[GCMS]	Air	Outdoor	Outdoor	1000L	F/2
Tetraselmis sp.	-	0.1	18.6	Huerlimann et al. (2010)[12h]	Air	12L:12D	250 μmol photons m^{-2} s^{-1}	10 L	F/2
Tetraselmis sp.	-	-	43.4	Rodolfi et al. (2009)[24h, CO2]	Air/CO_2 (95/5)	24L:0D	100 μmol PAR photons m^{-2} s^{-1}	100 mL	F
Tetraselmis sp.	-	-	10.7	Patil et al. (2007)[GCMS, 24h, CO2]	Air & CO_2	24L:0D	11 W	1.8 L	Z8/2
T. chui	0.35	1.0	1.5	This study[GCMS, AG]	Agitation	12L:12D	120 μmol photons m^{-2} s^{-1}	20 mL	F
T. chui	-	-	27.0	Rodolfi et al. (2009)[24h, CO2]	Air/CO_2 (95/5)	24L:0D	100 μmol PAR photons m^{-2} s^{-1}	100 mL	F
T. suecica	0.37	0.5	1.5	This study[GCMS, AG]	Agitation	12L:12D	120 μmol photons m^{-2} s^{-1}	20 mL	F
T. suecica	-	-	36.4	Rodolfi et al. (2009)[24h, CO2]	Air/CO_2 (95/5)	24L:0D	100 μmol photons m^{-2} s^{-1}	100 mL	F
T. suecica	0.30	-	-	Lee et al. (2010)[12h]	Air	12L:12D	136.3 μmol photons m^{-2} s^{-1}	15 L	F/2
T. tetrathele	-	1.3	-	de la Pena & Villegas (2005)[12h]	Air	14L:10D	100 μmol photons m^{-2} s^{-1}	1 L	F
D. salina	0.30	0.8	4.8	This study[GCMS, AG]	Agitation	12L:12D	120 μmol photons m^{-2} s^{-1}	20 mL	F
D. salina	-	0.4	-	Raja et al. (2007)[AG]	-	12L:12D	30 μmol photons m^{-2} s^{-1}	100 mL	De Walne
D. salina	-	0.3	-	Garcia et al. (2007)[24h]	Air	12L:12D	80 μmol photons m^{-2} s^{-1}	1 L	J
D. salina	-	-	33.5	Takagi et al. (2006)[24h, CO2]	Air/CO_2 (97/3)	24L:0D	150 μmol photons m^{-2} s^{-1}	500 mL	Modified NORO
D. viridis	-	0.2	-	Garcia et al. (2007)[24h]	Air	12L:12D	80 μmol photons m^{-2} s^{-1}	1 L	J
C. muelleri	0.35	0.7	3.3	This study[GCMS, AG]	Agitation	12L:12D	120 μmol photons m^{-2} s^{-1}	20 mL	F
Chaetoceros muelleri			21.8	Rodolfi et al. (2009)[24h, CO2]	Air/CO_2 (95/5)	24L:0D	100 μmol photons m^{-2} s^{-1}	100 mL	F
C. calcitrans	0.34	0.6	3.2	This study[GCMS, AG]	Agitation	12L:12D	120 μmol photons m^{-2} s^{-1}	20 mL	F

C. calcitrans	-	-	17.6	Rodolfi et al. (2009)[24h, C02]	Air/CO_2 (95/5)	24L:0D	100 µmol photons m^{-2} s^{-1}	100 mL	F
C. calcitrans	0.27	-	-	Lee et al. (2010)[12h]	Air	12L:12D	136.3 µmol photons m^{-2} s^{-1}	15 L	F/2
Chaetoceros sp.	0.74	-	16.8	Renaud et al. (2002)* [12h]	Air & CO_2	12L:12D	80 µmol photons m^{-2} s^{-1}	1.5 L	F/2
C. wighamii	-	1.6	-	Araujo & Garcia (2004)[12h]	Air	12L:12D	530 µmol photons m^{-2} s^{-1}	6 L	F/2
I. galbana	0.35	0.6	2.0	This study[GCMS, AG]	Agitation	12L:12D	120 µmol photons m^{-2} s^{-1}	20 mL	F
Isochrysis sp.	0.81	-	24.9	Renaud et al. (2002)* [12h]	Air & CO_2	12L:12D	80 µmol photons m^{-2} s^{-1}	1.5 L	F/2
Isochrysis sp.	-	0.2	12.7	Huerlimann et al. (2010)[12h]	Air	12L:12D	250 µmol photons m^{-2} s^{-1}	10 L	F/2
Isochrysis sp.	-	-	37.7	Rodolfi et al. (2009)[24h, C02]	Air/CO_2 (95/5)	24L:0D	100 µmol photons m^{-2} s^{-1}	100 mL	F
I. galbana	0.10	-	-	Lee et al. (2010)[12h]	Air	12L:12D	136.3 µmol photons m^{-2} s^{-1}	15 L	F/2
I. galbana	-	-	12.4	Patil et al. (2007)[GCMS, 24h, CO2]	Air & CO_2	24L:0D	11 W	1.8 L	Z8/2
P. lutheri	0.48	0.8	2.0	This study[GCMS, AG]	Agitation	12L:12D	120 µmol photons m^{-2} s^{-1}	20 mL	F
P. lutheri	-	-	50.2	Rodolfi et al. (2009)[24h, C02]	Air/CO_2 (95/5)	24L:0D	100 µmol photons m^{-2} s^{-1}	100 mL	F
P. salina	0.45	0.9	2.1	This study[GCMS, AG]	Agitation	12L:12D	120 µmol photons m^{-2} s^{-1}	20 mL	F
P. salina	-	1.8	-	Emdadi & Berland (1989)[24h]	Air	24L:0D	50 µE m^{-2} s^{-1}	8 L	-
P. salina			49.4	Rodolfi et al. (2009)[24h, C02]	Air/CO_2 (95/5)	24L:0D	100 µmol photons m^{-2} s^{-1}	100 mL	F
P. viridis	-	0.7	-	Xu et al. (2008)[24h]	Air	24L:0D	120 µmol photons m^{-2} s^{-1}	10 L	Personal
Pavlova sp.	-	-	21.7	Patil et al. (2007)[GCMS, 24h, CO2]	Air & CO_2	24L:0D	11 W	1.8 L	Z8/2
Chlorella sp. BR2	0.34	0.9	3.9	This study[GCMS, AG]	Agitation	12L:12D	120 µmol photons m^{-2} s^{-1}	20 mL	F
Chlorella sp.	-	0.5	7.1	Chen et al. (2010)[AG]	Agitation	12L:12D	9 W m^{-2}	1 L	Personal
C. vulgaris	0.14	-	20.2	Converti et al. (2009)[24h, CO2]	Air/CO_2 (95/5)	24L:0D	100 µmol photons m^{-2} s^{-1}	100 mL	F
Chlorella sp.	-	-	42.1	Rodolfi et al. (2009)[24h, C02]	Air/CO_2 (95/5)	24L:0D	100 µmol photons m^{-2} s^{-1}	100 mL	F
C. sorokiana	-	-	44.7	Rodolfi et al. (2009)[24h, C02]	Air/CO2 (95/5)	24L:0D	25 µmol photons m^{-2} s^{-1}	2L	Watanabe
C. sorokiana	-	0.6	1.0	Illman et al. (2000)[24h, CO2]	Inc: Air & CO_2	24L:0D	70 µE m^{-2} s^{-1}	2 L	F/2
C. vulgaris	-	1.0	5.3	Illman et al. (2000)[24h, CO2]	Air/CO2 (95/5)	24L:0D	25 µmol photons m^{-2} s^{-1}	2 L	Watanabe

*Calculated total lipid content (µg mL^{-1})

[GCMS]Values obtained by GCMS

[24h]Culutures grown with 24 h light and air

[12h]Culutures grown with 12 h light and air

[C02]Cultures grown with air supplemented with CO_2

[AG]Cultures grown with agitation
